# Molecular epidemiology of *Bartonella quintana* endocarditis in patients from Israel and Eastern Africa

**DOI:** 10.1186/s12879-023-08099-x

**Published:** 2023-03-07

**Authors:** Shingo Sato, Lev Shapira, Diana Tasher, Soichi Maruyama, Michael Giladi

**Affiliations:** 1 Laboratory of Veterinary Public Health, Department of Veterinary Medicine, College of Bioresource Sciences, Nihon University, 1866 Kameino, Fujisawa, Kanagawa 252-0880 Japan; 2 The Bernard Pridan Laboratory for Molecular Biology of Infectious Diseases, Tel Aviv Sourasky Medical Center, 6 Wiezmann Street, 64239 Tel Aviv, Israel; 3 Pediatric Infectious Disease Unit, Wolfson Medical Center, Holon, Israel and the Sackler Faculty of Medicine, Tel Aviv University, Tel Aviv, Israel; 4 Infectious Disease Unit, Tel Aviv Sourasky Medical Center and the Sackler Faculty of Medicine, Tel Aviv University, Tel Aviv, Israel

**Keywords:** *Bartonella quintana*, Endocarditis, ST, MLST, Israel, Eastern Africa

## Abstract

**Background:**

*Bartonella quintana* is an important cause of culture-negative endocarditis. Although humans have been considered as its only reservoir, recent studies showed that macaque species are also reservoirs of *B. quintana*. Based on multi-locus sequence typing (MLST) *B. quintana* strains have been classified into 22 sequence types (STs), with 7 STs exclusively found in humans. Data regarding the molecular epidemiology of *B. quintana* endocarditis is limited to only 3 STs identified in 4 patients from Europe and Australia. We studied *B. quintana* endocarditis acquired in Eastern Africa or Israel to investigate the genetic diversity and clinical relatedness of *B. quintana* from distinct geographic regions.

**Methods:**

Eleven patients with *B. quintana* endocarditis, 6 from Eastern Africa and 5 from Israel, were studied. DNA was extracted from cardiac tissue or blood specimens and analyzed by MLST based on 9 genetic loci. An evolutionary relationship between STs was visualized by a minimum spanning tree. A phylogenetic tree was constructed with the concatenated sequences (4271 bp) of the 9 loci using the maximum-likelihood method.

**Results:**

Six strains were classified into previously described STs while 5 strains were identified for the first time and classified into new STs 23–27 which clustered with the previously reported STs 1–7 from human strains found in Australia, France, Germany, the USA, Russia, and the former Yugoslavia, without indication of geographical structuring. ST2 was the most prevalent ST, found in 5 of 15 patients with endocarditis (33.3%). ST26 appears to be a primary founder of the human lineage.

**Conclusions:**

The new and previously reported human STs form a single human lineage, clearly separated from the other 3 *B. quintana* lineages of cynomolgus, rhesus, and Japanese macaques. From evolutionary perspectives, these findings support the assumption that *B. quintana* has co-evolved with host species to form a host-speciation pattern. ST26 is suggested herein as a primary founder of the human lineage and may be key to explore where *B. quintana* had first originated; ST2 is a dominant genetic type associated with *B. quintana* endocarditis. To confirm these findings, additional worldwide molecular epidemiological studies are required.

## Introduction

*Bartonella quintana* is a fastidious Gram-negative bacterium transmitted among humans by the body louse *Pediculosis humanus humanus* and known to cause trench fever, chronic bacteremia, bacillary angiomatosis and endocarditis. Trench fever was first described in Europe in 1915, during World War I [1], and later during World War II [2, 3]. However, recent paleomicrobiological studies have provided evidence that *B. quintana* has been associated with human infection for 4000 years [4]. After an interval of several decades where trench fever almost disappeared, small outbreaks of *B. quintana* chronic bacteremia have reemerged in HIV-uninfected people living in urban areas. Homelessness, alcoholism and louse infestation were identified as risk factors [1, 3, 5, 6].

For years, humans have been considered as the only reservoir of *B. quintana*, however, recent studies have identified the non-human primate macaques as natural hosts of *B. quintana* [7, 8]. Based on multi-locus sequence typing (MLST), *B. quintana* strains have been classified into 22 sequence types (STs): STs 1–7 exclusively found in humans [9], STs 8–14 and STs 15–21 in cynomolgus macaque and in rhesus macaque strains from China [7], respectively, and ST22 in Japanese macaque strains in Japan [8].

*Bartonella* species is recognized as an important cause of culture-negative endocarditis, accounting for 2–40% of cases, with *B. quintana* responsible for the majority of *Bartonella* endocarditis cases [10–16]. Data regarding the molecular epidemiology of *B. quintana* strains causing endocarditis, however, is limited to the description of only 3 STs (STs 1, 2, and 7) identified in 4 endocarditis patients from Europe and Australia [9].

In the present study, we applied *B. quintana*-specific MLST technique to 11 *Bartonella* endocarditis patients diagnosed in Israel, who have presumably acquired the infection in Eastern Africa or in Israel, to investigate the genetic diversity and clinical relatedness of *B. quintana* strains identified in endocarditis patients from distinct geographic regions.

## Materials and methods

### Patients and clinical specimens

Eleven patients with definite endocarditis, as defined in the modified Duke criteria [17], were included in this study. All patients have undergone valve surgery for endocarditis-associated valve dysfunction or the presence of intracardiac abscess; 5 patients arrived from Eastern Africa for cardiothoracic care at Wolfson Medical Center in Israel through the Save a Child’s Heart fund activity, 1 patient immigrated from Ethiopia to Israel while being severely ill with endocarditis, and 5 patients have been Israeli residents for several years prior to diagnosis. PCR of cardiac specimens (10 patients) and of a *B. quintana* blood culture isolate (1 patient) followed by DNA sequencing was performed as previously described [18]. Relevant patient characteristics are summarized in Table 1. Patients no. 1–5 have been described before [19, 20].

**Table 1:** Characteristics of 11 patients with *B. quintana *endocarditis

Patient no.	Age-years / sex	Country of presumed acquisition of *B. quintana *infection	Clinical specimens	Year of diagnosis	Strain designation
1	9/F	Ethiopia	Cardiac tissue	2014	EA1
2	7/F	Ethiopia	Cardiac tissue	2014	EA3
3	12/F	Ethiopia	Cardiac tissue	2014	EA4
4	16/F	Ethiopia	Cardiac tissue	2015	EA5
5	20/M	Ethiopia	Cardiac tissue	2003	EA6
6	11/M	Ruanda	Cardiac tissue	2019	EA7
7	28/M	Israel	Cardiac tissue	2014	EA2
8	32/M	Israel	Cardiac tissue	2003	ISEA
9	75/M	Israel	Cardiac tissue	2015	IS2
10	65/M	Israel	Cardiac tissue	2016	IS3
11	67/M	Israel	Isolate from blood	2005	IS1/Bq-TA7

*F* female, *M* male

### MLST analysis of *B. quintana* DNA

Genomic DNA was extracted from cardiac tissues of 10 patients using the QIAamp DNA minikit (Qiagen, Valencia, CA) and from a *B. quintana* blood culture isolate of 1 patient using E.Z.N.A. bacterial DNA kit (Omega Bio-Tek), and then kept at − 20 °C until further analysis. All DNA samples were subsequently analyzed by *B. quintana*-specific MLST scheme which has been developed based on 9 genetic loci (*atpF*, *bqtR*, *ftsZ*, *gap*, *gltA*, *groEL*, *nlpD*, *ribE*, *rpoB*) [9]. PCR conditions for all loci were as follows: initial denaturation at 94 °C for 5 min, followed by 35 cycles (94 °C for 1 min, 48 °C for 30 s, and 72 °C for 45 s), and a final extension step at 72 °C for 6 min. Genomic DNA from *B. quintana* Toulouse strain (CIP 103739) and nuclease-free water were used for positive and negative controls, respectively. Nucleotide sequences of the PCR products were determined for both strands and repeated as necessary (DNA Sequencing Unit at the G.S. Wise Faculty of Life Sciences, Tel Aviv University, Israel). The nucleotide sequences of each locus were compared with MLST data previously reported [7–9] using a genomic analysis software GENETYX ver.15 (GENETYX Corp., Tokyo, Japan) and were assigned allelic numbers. New allelic combinations identified in the present study were assigned to new STs.

An evolutionary relationship between the new STs and the previously reported STs (1 to 22) was visualized by a minimum spanning tree constructed based on goeBURST algorithm implemented in PHYLOViZ 2.0 [21], which allows phylogenetic inference and data visualization. A lineage was defined as the same group of STs sharing identical alleles at 7 or more of the 9 loci. A phylogenetic tree was constructed with the concatenated sequences (4271 bp) of the 9 loci in each ST by using the maximum-likelihood method in MEGA X [22]. *B. henselae* Houston-1^T^ was set as an outgroup in the tree. Substitution model was Tamura 3-parameter model (T92) and gap/missing data were treated by complete deletion. Bootstrap analysis was conducted with 1000 replicates.

## Results

**Fig. 1:**
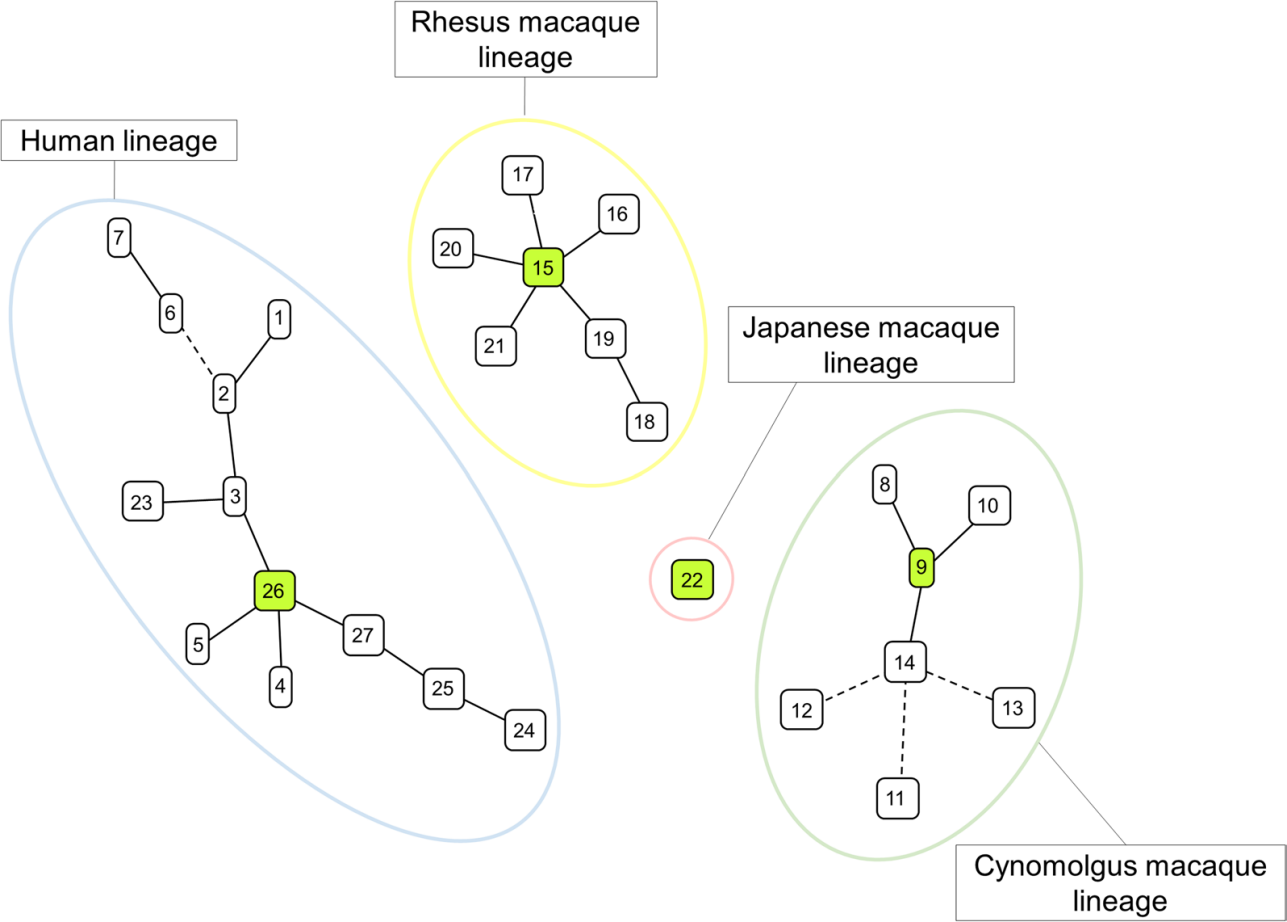
Minimum spanning tree constructed from allelic profiles of STs 1 to 27 of *B. quintana* strains by goeBURST in PHYLOViZ 2.0. A lineage was defined as the same group of STs sharing identical alleles at 7 or more of the 9 loci. Black line and black dashed line indicate 1 and 2 differences of the locus between two STs, respectively. Green color square shows a founder in each lineage. Color circles show 4 lineages classified by host species

**Fig. 2:**
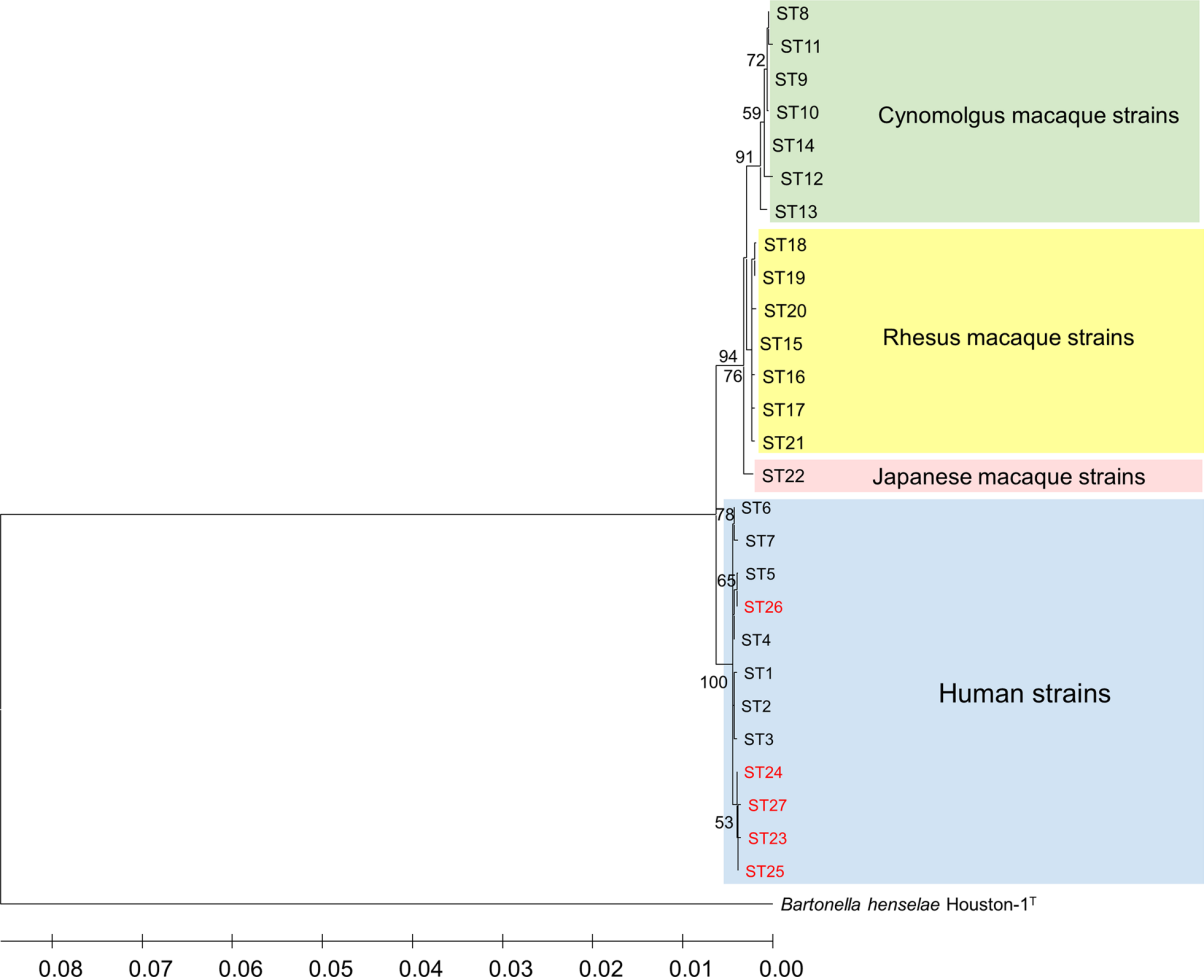
Phylogenetic tree constructed from the 9 loci-concatenated sequences of STs 1 to 27 of *B. quintana* strains. The tree was constructed from the concatenated sequences (4271 bp) of the 9 loci used for MLST analysis by using the maximum-likelihood method based on the Tamura 3-parameter model in MEGA X [22]. The *B. quintana* strains from humans (STs 1 to 7 and STs 23 to 27), cynomolgus macaques (STs 8 to 14), rhesus macaques (STs 15 to 21), and Japanese macaques (ST22) were included in the tree. Colored rectangles show 4 groups classified by host species. The scale bar indicates estimated evolutionary distance. Bootstrap values were obtained with 1000 replicates. Only bootstrap replicates > 50% are noted. *B. henselae* Houston-1^T^ was set as an outgroup in the tree

The 9 genetic loci of *B. quintana* were detected in all patient-derived specimens. Strains EA1, EA3, and EA6, strain IS3, and strains IS1/Bq-TA7 and IS2 were classified into the previously described STs 2, 4, and 6, respectively. Strains EA2, EA4, EA5, ISEA, and EA7 were identified for the first time and classified into 5 new STs 23–27, resulting in discrimination of human strains into a total of 12 STs, including 8 endocarditis-related STs (Table 2). In a previous study by Arvand et al. [9], 4 of the 16 *B. quintana* strains were identified in clinical specimens of patients with endocarditis: ST1 in one strain (Jouhanneau), ST2 in two strains (strains UR.BQ.TIE 326 and HROEH), and ST7 in one strain (Adelaide 1300/02). Together with the current study, there are 15 patients with *B. quintana* endocarditis analyzed by MLST, with ST2, being the most prevalent, found in 5 patients (33.3%). The new endocarditis-related STs 23–27 clustered with the previously reported STs 1–7 from humans infected with *B. quintana* as supported by the minimum spanning tree and the phylogenetic tree, constructed based on the 9 loci (Figs. 1, 2). Moreover, ST26, found in strain ISEA, appears to be a primary founder in the human lineage, as it is surrounded by STs 3, 4, 5, and 27, each differs from ST26 by only a single locus (Fig. 1). Strain ISEA was detected in 2003 in the aortic valve of a 32-year-old male (patient no. 8, Table 1), who was born in Ethiopia and immigrated to Israel at the age of 13. Therefore, *B. quintana* strain ISEA showing ST26 has most likely been acquired in Israel.

**Table 2:** Allelic profiles and sequence types (ST) identified in this study

Strain designation	*atpF*	*bqtR*	*ftsZ*	*gap*	*gltA*^a^	*groEL*	*nlpD*	*ribE*	*rpoB*	ST^b^
EA1, EA3, EA6	1	1	1	1	1	1	1	1	2	2
IS3	1	1	1	1	1	2	1	1	2	4
IS1/Bq-TA7, IS2	1	1	2	1	1	3	1	1	2	6
EA2	1	1	1	1	5	1	2	1	2	23
EA4	1	1	1	1	5	3	1	1	2	24
EA5	1	1	1	1	5	3	2	1	2	25
ISEA	1	1	1	1	1	2	2	1	2	26
EA7	1	1	1	1	5	2	2	1	2	27

^a^The *gltA* of STs 23, 24, 25, and 27 shows a new allelic number, the nucleotide sequence of which was registered in the GenBank/EMBL/DDBJ under accession number LC667375

^b^STs 23 to 27 are newly reported in the present study

## Discussion

In the current study, we have analyzed genomic DNA of 11 *B. quintana* stains from patients with endocarditis by using MLST and identified 5 new STs which clustered with the previously reported STs 1–7 from humans infected with *B. quintana.* Although Arvand et al. [9] presented a phylogenetic relationship among these 7 STs determined by eBURST, the STs were divided into two clonal complexes and a singleton, which was not qualified for a definition of a single group. The new STs described herein were found to provide the missing links to form a single human lineage, clearly separated from the other 3 lineages of *B. quintana* strains of cynomolgus macaque, rhesus macaque, and Japanese macaque. From evolutionary perspectives, these findings further support the assumption that *B. quintana* strains have co-evolved with host species to form a host-speciation pattern.

Among the new STs, ST26 found in strain ISEA is suggested herein as a primary founder of the human lineage, rather than ST2 suggested previously [9]. Of note, in previous reports [7–9], ST5, identified in strain Fuller^T^ isolated in 1945 from a trench fever patient in the former Yugoslavia, was assigned as a singleton without any phylogenetic link to other STs since it differed in 2 or more alleles from all other STs. The phylogenetic analyses of the present study showed that of all the STs identified in Australia, France, Germany, Russia, and the USA, ST26 found in strain ISEA is the most closely related to ST5 (Figs. 1, 2), a finding that further supports ST26 as being a group founder. In addition, we have shown that ST2 is the most frequent ST causing endocarditis in humans. Of interest is the fact that the 15 *B. quintana* strains detected and analyzed by MLST hitherto in patients with endocarditis were identified in geographically diverse countries in 5 different continents, Europe, America and Australia previously reported [9] as well as Africa and Asia (Middle East) firstly reported here, without indication of geographical structuring.

To clarify whether ST26 is a veritable primary founder in human-derived *B. quintana* strains, substantiate an epidemiological association between ST2 and endocarditis, and explore where *B. quintana* had first originated, more molecular epidemiological studies are needed to be conducted worldwide.

## Data Availability

The *gltA* sequence of STs 23, 24, 25, and 27 was registered in the GenBank/EMBL/DDBJ under accession number LC667375.

## Abbreviations

MLST: Multi-locus sequence typing
ST: Sequence type

## References

[1] Foucault C, Brouqui P, Raoult D (2006). *Bartonella **quintana* characteristics and clinical management. Emerg Infect Dis.

[2] Kostrzewski J (1949). The epidemiology of trench fever. Bull Acad Pol Sci.

[3] Maurin M, Raoult D (1996). *Bartonella* (Rochalimaea) *quintana* infections. Clin Microbiol Rev.

[4] Drancourt M, Tran-Hung L, Courtin J, Lumley H, Raoult D (2005). *Bartonella **quintana* in a 4000-year-old human tooth. J Infect Dis.

[5] Spach DH, Kanter AS, Dougherty MJ, Larson AM, Coyle MB, Brenner DJ (1995). *Bartonella* (Rochalimaea) *quintana* bacteremia in inner-city patients with chronic alcoholism. N Engl J Med.

[6] Drancourt M, Mainardi JL, Brouqui P, Vandenesch F, Carta A, Lehnert F (1995). *Bartonella* (*Rochalimaea*) *quintana* endocarditis in three homeless men. N Engl J Med.

[7] Li H, Bai JY, Wang LY, Zeng L, Shi YS, Qiu ZL (2013). Genetic diversity of *Bartonella **quintana* in macaques suggests zoonotic origin of trench fever. Mol Ecol.

[8] Sato S, Kabeya H, Yoshino A, Sekine W, Suzuki K, Tamate HB (2015). Japanese macaques (*Macaca **fuscata*) as natural reservoir of *Bartonella **quintana*. Emerg Infect Dis.

[9] Arvand M, Raoult D, Feil EJ (2010). Multi-locus sequence typing of a geographically and temporally diverse sample of the highly clonal human pathogen *Bartonella **quintana*. PLoS ONE.

[10] Houpikian P, Raoult D (2005). Blood culture-negative endocarditis in a reference center: etiologic diagnosis of 348 cases. Medicine (Baltimore).

[11] Murdoch DR, Corey GR, Hoen B, Miró JM, Fowler VG, Bayer AS (2009). Clinical presentation, etiology, and outcome of infective endocarditis in the 21st century: the international collaboration on endocarditis-prospective cohort study. Arch Intern Med.

[12] Fournier PE, Thuny F, Richet H, Lepidi H, Casalta JP, Arzouni JP (2010). Comprehensive diagnostic strategy for blood culture-negative endocarditis: a prospective study of 819 new cases. Clin Infect Dis.

[13] Lamas CC, Fournier PE, Zappa M, Brandão TJ, Januário-da-Silva CA, Correia MG (2016). Diagnosis of blood culture-negative endocarditis and clinical comparison between blood culture-negative and blood culture-positive cases. Infection.

[14] Okaro U, Addisu A, Casanas B, Anderson B (2017). *Bartonella* species, an emerging cause of blood-culture-negative endocarditis. Clin Microbiol Rev.

[15] Fournier PE, Gouriet F, Casalta JP, Lepidi H, Chaudet H, Thuny F (2017). Blood culture-negative endocarditis: improving the diagnostic yield using new diagnostic tools. Medicine (Baltimore).

[16] Fida M, Dylla BL, Sohail MR, Pritt BS, Schuetz AN, Patel R (2019). Role of prolonged blood culture incubation in infective endocarditis diagnosis. Eur J Clin Microbiol Infect Dis.

[17] Li JS, Sexton DJ, Mick N, Nettles R, Fowler VG, Ryan T (2000). Proposed modifications to the Duke criteria for the diagnosis of infective endocarditis. Clin Infect Dis.

[18] Shapira L, Rasis M, Binsky Ehrenreich I (2021). Laboratory diagnosis of 37 cases of *Bartonella* endocarditis based on enzyme immunoassay and real-time PCR. J Clin Microbiol.

[19] Tasher D, Raucher-Sternfeld A, Tamir A, Giladi M, Somekh E (2017). *Bartonella **quintana*, an unrecognized cause of infective endocarditis in children in Ethiopia. Emerg Infect Dis.

[20] Goldstein H, Saliba R, Elias M, Zlotnik A, Raz R, Giladi M (2005). *Bartonella **quintana* endocarditis in east Africa. Eur J Intern Med.

[21] Nascimento M, Sousa A, Ramirez M, Francisco AP, Carrico JA, Vaz C (2017). PHYLOViZ 2.0: providing scalable data integration and visualization for multiple phylogenetic inference methods. Bioinformatics.

[22] Kumar S, Stecher G, Li M, Knyaz C, Tamura K (2018). MEGA X: Molecular evolutionary genetics analysis across computing platforms. Mol Biol Evol.

